# Does education level affect the efficacy of a community based salt reduction program? - A post-hoc analysis of the China Rural Health Initiative Sodium Reduction Study (CRHI-SRS)

**DOI:** 10.1186/s12889-016-3454-6

**Published:** 2016-08-11

**Authors:** Xin Wang, Xian Li, Ilonca Vaartjes, Bruce Neal, Michiel L. Bots, Arno W. Hoes, Yangfeng Wu

**Affiliations:** 1 Julius Center for Health Sciences and Primary Care, University Medical Center Utrecht, Utrecht, The Netherlands; 2 The George Institute for Global Health at Peking University Health Science Center, Level 18, Tower B, Horizon Tower, No. 6 Zhichun Rd, Haidian District, Beijing, 100088 People’s Republic of China; 3 The George Institute for Global Health, Australia, Sydney, Australia

**Keywords:** Salt intake, Education, China, Cluster-randomized controlled trial

## Abstract

**Background:**

Whether educational level influences the effects of health education is not clearly defined. This study examined whether the impact of a community-based dietary salt reduction program was affected by the level of education of participants.

**Methods:**

The China Rural Health Initiative Sodium Reduction Study (CRHI-SRS) was a cluster-randomized controlled trial conducted in 120 villages from five Northern Chinese provinces. The intervention comprised a village-wide health education program and availability of salt substitute at village shops. 24-h urine samples were collected among 1903 participants for primary evaluation of the intervention effect. A post-hoc analysis was done to explore for heterogeneity of intervention effects by education level using generalized estimating equations. All models were adjusted for age, sex, body mass index and province.

**Results:**

Daily salt intake was lower in intervention than in control at all educational levels with no evidence of a difference in the effect of the intervention across different levels of education. *P* value for the interaction term between education level and the intervention was 0.35. There was likewise no evidence of an interaction for effects of the intervention on potassium intake (*p* = 0.71), the sodium to potassium ratio (*p* = 0.07), or knowledge and behaviors related to salt (all *p* > 0.05).

**Conclusions:**

The study suggests that the effects of the intervention were achieved regardless of the level of education and that the intervention should therefore be broadly effective in rural Chinese populations.

**Trial registration:**

The trial was registered with clinicaltrial.gov (NCT01259700).

## Background

Cardiovascular disease (CVD) is a major health concern in China. The number of patients with hypertension is about 266 million [1] and high blood pressure is one of the most prevalent modifiable risk factors for CVD. Reducing body weight, alcohol consumption and salt intake are important strategies for reducing blood pressure and should prevent much vascular disease burden [2, 3]. Benefits are likely to be additive to those achieve with pharmacological interventions and particularly important in lower income settings where uptake of drug therapies can be limited [4, 5].

In western countries, the average salt intake is typically between 7 and 12 g per day [6] and most dietary salt derives from processed and restaurant foods [7]. By contrast, in rural China, daily salt intake can be 12 to 15 g per day [7] and the major dietary source is salt added during home cooking and for seasoning at the table [8]. Although there remains debate about the merits of reducing salt intake to very low levels [9], for countries like China with very high levels of consumption, there is a broad consensus that benefit will ensue.

The China Rural Health Initiative Sodium Reduction Study (CRHI-SRS) was recently completed in rural China [10] and the low intensity community-based salt reduction program implemented in that study reduced salt intake by about 0.8 g per day [11]. There may be as many as 54 million Chinese who are illiterate and three quarters of these individuals live in rural areas [12]. Lower levels of education have been associated with less good adherence to medications, knowledge of disease and self-care [13–16]. As such, it is possible that the less educated participants in the CRHI-SRS gained less benefit than their better educated counterparts. This is important to know because health promotion programs targeting salt need to meet the needs of all population groups. To explore this hypothesis, we investigated whether the effects of CRHI-SRS community-based salt reduction program differed between individuals with different levels of education.

## Methods

### Study design and participants

The CRHI-SRS was a large-scale cluster-randomized controlled trial. Details of the rationale and study design have been described previously [10]. The trial was registered with clinicaltrial.gov (NCT01259700). In brief, the trial was conducted in northern rural China between May 2011 and November 2012. Two counties were selected from each of 5 provinces (Hebei, Liaoning, Shanxi, Shaanxi and the Ningxia Autonomous Region), 12 townships from each county and 1 village from each township, making a total of 120 villages. Townships were randomized centrally in a 1:1 ratio with stratification by county.

### Intervention and control strategies

The sodium reduction intervention comprised community-based health education activities and making salt substitute available at village shops [10]. The education activities were designed on the basis of the health belief model aiming to achieve population-wide salt reduction. These activities were delivered by the township health educators with assistance from the village council and the village doctors through public lectures, posters, displays and distribution of promotional materials to every family including calendars, pamphlets, salt jar stickers, and special interactive education sessions targeted towards individuals at elevated risk of vascular diseases. The salt substitute was made available for purchase in all intervention villages and promoted through the health education activities. Half of the intervention villages were randomized to receive a price subsidy where residents in the villages were provided with coupons that enabled the purchase of salt substitute at the same price as usual salt. The key messages delivered through the health education activities included: harms of high salt intake, the recommendations of the Chinese Society of Nutrition on daily salt intake for adults, methods and skills to reduce salt intake. The instructions on salt substitute use put emphasis on not over consuming and reminded individuals using potassium-sparing diuretics or with chronic renal disease not to use the salt substitute. These instructions were printed and delivered together with the salt substitute at the point of sale. Control villages continued their usual practices without any of the salt reduction efforts described above [10].

### Data collection

At the end of the intervention, twenty adults, age- and sex-stratified, were drawn from each village for the evaluation survey except for one village in the control group that was lost to follow-up due to the urbanization of the area. The survey consisted of an interviewer-administered questionnaire, measurement of blood pressure, height and weight, and the collection of a 24-h urine sample. Years of school education were recorded and participants were divided into four groups aligned with usual Chinese educational system classifications - “no school” which corresponds to illiterate; “low education” which corresponds to 1–6 years of schooling (elementary school); “middle education” which corresponds to 7–9 years of schooling (middle school) or; “high education” which corresponds to more than 9 years of education (high school and above). Blood pressure was measured twice on one occasion with participants in the seated position after 5 min of rest using an automated electronic sphygmomanometer (Omron Interllisense HEM 7301 IT). The mean of these two measurements was used in the analysis. A 24-h urine sample was collected using standard methods [10] and the urine volume was noted. An aliquot was shipped to a central lab for assay of urine sodium, potassium and creatinine. Urine collections were excluded if participants reported missing more than one void, a collection period less than 22 h or longer than 26 h or suspected spillage of more than 10 % of the total volume. Any 24-h urine collection of < 500 ml or > 6000 ml was also excluded as were samples with urinary creatinine < 4.0 mmol/day or > 25 mmol/day for women or urinary creatinine < 6.0 mmol/day or > 30 mmol/day for men [1].

For each individual, the 24-h sodium excretion (mmol/day) was calculated as the concentration of sodium (mmol/L) multiplied by the urinary volume (L/day). Similarly, the 24-h potassium excretion (mmol/day) was calculated. The sodium to potassium ratio (Na/K ratio) was calculated as 24-h sodium excretion divided by 24-h potassium excretion value. Sodium values in mmol/day were converted to salt in grams per day as follows: 1 mmol sodium = 23 mg sodium.

### Statistical analysis

Characteristics of participants were summarized as proportions or means with corresponding uncertainty intervals in strata defined by level of education. The main outcomes were urinary sodium, urinary potassium and the sodium to potassium ratio. Knowledge and behaviors related to salt were also examined as intermediate markers that might be helpful in understanding the modifying effects of educational level on the fidelity of the intervention. We used generalized estimating equations (GEE), which accounted for clustering within regions and villages to estimate the intervention effects across different levels of education. An interaction term (education* intervention) was included in the models and a *p*-value of < 0.05 was considered statistically significant. We controlled for the potential confounding factors of age, sex, body mass index and province using multivariable adjustment. All analyses should be considered as exploratory post-hoc analyses of the trial and were performed using SPSS 20.0 for Windows (SPSS Inc., Chicago, IL, USA).

### Ethical considerations

The project was reviewed and approved by the institutional review boards of both Peking University in China and Duke University in the US. Consent for participation in CRHI-SRS was sought at the cluster level and the individual level. Community consent of the study was obtained through a consultation process involving the government authorities at provincial, county and township levels and the village leaders. Individual consent for participations was obtained from all persons selected in outcome surveys. Participants identified during the survey that require medical attention were referred to existing services in line with usual practice. Data collected in the study was kept in a password protected system and only de-identified data was used for analyses.

## Results

There were 1,903 participants that provided eligible urine samples who were included in the final analysis - 975 from intervention villages and 928 from control villages (Table 1). The mean age of the participants was 54.5 years and 50.4 % were women. Characteristics were not different between intervention and control villages [11] although there were substantial differences across educational groups (Table 2). People with more years of education were younger and people with less years of education were more likely to be female. There were also large regional differences in educational levels with only 4.6 % participants from Ningxia province having more than 9 years of education while the corresponding figure was 13.4 % for participants from Hebei province.

**Table 1:** General characteristics and outcomes of the sodium reduction program for intervention and control villages obtained at the end of the study period

	Intervention *N* = 975	Control *N* = 928
*Demographics*		
Age (years)	55.1 ± 13.8	53.9 ± 14.1
Female (%)	50.7	50.2
Formal education (%)		
0 years	16.5	18.1
1–6 years	38.4	34.8
7–9 years	37.4	36.7
>9 years	7.7	10.4
*Behaviors*		
Current smoker (%)	32.2	29.7
Drink alcohol (%)^a^	23.5	25.9
*Disease history (%)*		
Coronary heart disease	8.6	9.3
Stroke	4.3	4.8
Diabetes	4.2	4.3
Hypertension	26.1	28.2
*Physical examination*		
BMI (kg/m^2^)	24.5 ± 3.5	24.8 ± 3.5
*Outcomes*		
Urinary Na (mmol/day)	236.7 ± 96.6	250.5 ± 93.9
Urinary K (mmol/day)	52.7 ± 25.3	45.4 ± 18.9
Urinary Na/K ratio	5.2 ± 3.1	6.1 ± 2.5
SBP (mmHg)	140.4 ± 21.2	141.3 ± 22.1
DBP (mmHg)	86.0 ± 13.3	86.3 ± 13.6

*SBP* systolic blood pressure; *DBP* diastolic blood pressure; *BMI* body mass index. ^a^Drink alcohol is defined as having any type of alcohol in the three months prior to survey

**Table 2:** General characteristics in subgroups of different educational levels, obtained at the end of the study period

Education	0 yrs (*n* = 329)	1–6 yrs (*n* = 697)	7–9 yrs (*n* = 704)	>9 yrs (*n* = 171)	*P* values
Age (years)	63.8 ± 9.6	59.5 ± 10.7	47.7 ± 13.5	44.8 ± 15.2	<0.001
Female (%)	75.4	52.5	41.5	30.4	<0.001
Current smoker (%)	14.9	28.6	38.5	41.5	<0.001
Drink alcohol^b^ (%)	7.6	20.9	33.5	36.5	<0.001
*Disease history (%)*	37.1	25.3	17.2	14.1	<0.001
Coronary heart disease	14.6	10.6	6.1	2.3	<0.001
Stroke	5.8	5.9	3.6	1.2	0.137
Diabetes	4.6	5.5	3.6	1.8	0.004
Hypertension	41.0	30.3	18.8	22.2	<0.001
*Physical examination*					
SBP (mmHg)^a^	146.9 ± 6.3	144.0 ± 7.0	136.4 ± 8.8	134.4 ± 9.9	<0.001
DBP (mmHg)^a^	86.4 ± 1.3	86.5 ± 1.6	85.5 ± 1.8	85.4 ± 2.0	<0.001
BMI (kg/m^2^)^a^	24.8 ± 0.2	24.7 ± 0.2	24.5 ± 0.3	24.4 ± 0.2	<0.001
*Province (%)*					<0.001
Liaoning	8.1	43.1	43.1	5.8	
Hebei	13.4	35.4	37.7	13.4	
Shanxi	13.6	41.5	37.4	7.5	
Shaanxi	9.1	36.1	42.6	12.2	
Ningxia	42.2	27.4	25.9	4.6	

^a^Continuous variables were adjusted for age and sex. ^b^Drink alcohol is defined as having any type of alcohol in the three months prior to survey

Among all educational groups, dietary sodium intake was numerically lower and dietary potassium intake was numerically higher in intervention than in control villages (Table 3). For instance, sodium intake in people with >9 years of education was 236 mmol/day in interventions villages and 269 mmol/day in control villages and for people with no formal education sodium intake was 206 mmol/day in intervention villages and 212 mmol/day in control villages. There was no evidence of a difference in the effects of intervention across different levels of education for sodium (*p* interaction 0.35), potassium (*p* = 0.71) or the sodium to potassium ratio (*p* = 0.07). Likewise, across all educational levels there was greater knowledge about the adverse effects of salt, concerns about salt in the diet and use of salt substitute in the intervention compared to control groups (Table 3) (all *p* interaction > 0.05). Finally, the use of salt substitute in villages where the price of salt substitute was subsidized was much greater than in those it was not subsidized but uptake was once again not different across educational levels (Table 3).

**Table 3:** Effect of sodium reduction strategy on dietary sodium, potassium and sodium to potassium ratio and knowledge and behaviors by different educational levels

Education	0 yrs	1–6 yrs	7–9 yrs	>9 yrs	
	Intervention vs. control	Intervention vs. control	Intervention vs. control	Intervention vs. control	*P* values*
Dietary sodium (mmol/day)	206.5 vs. 212.9	237.8 vs. 257.2	249.2 vs. 257.4	236.4 vs. 269.7	0.35
Dietary potassium (mmol/day)	50.2 vs. 42.3	53.5 vs. 48.3	52.6 vs. 44.9	53.2 vs. 43.1	0.71
Sodium to potassium ratio	4.7 vs. 5.5	5.3 vs. 5.9	5.3 vs. 6.3	5.1 vs. 6.6	0.07
Know salt is harmful	79 % vs. 70 %	82 % vs. 74 %	90 % vs. 81 %	94 % vs. 84 %	0.35
Know daily limit is ≤ 6 g/day	46 % vs. 11 %	51 % vs. 15 %	60 % vs. 23 %	65 % vs. 27 %	0.48
Know reducing salt lowers blood pressure	67 % vs. 48 %	67 % vs. 52 %	72 % vs. 58 %	73 % vs. 61 %	0.71
Concerned about salt in diet	79 % vs. 57 %	78 % vs. 61 %	75 % vs. 66 %	79 % vs. 57 %	0.27
Use salt substitute	53 % vs. 7 %	65 % vs. 7 %	65 % vs. 5 %	59 % vs. 7 %	0.17
	Subsidy vs. no subsidy	Subsidy vs. no subsidy	Subsidy vs. no subsidy	Subsidy vs. no subsidy	
Use salt substitute	72 % vs. 36 %	76 % vs. 50 %	80 % vs. 47 %	83 % vs. 31 %	0.51

^*^ *P* values stand for *p* values of interaction terms (education*intervention) in the generalized estimating equations

## Discussion

We identified no evidence of a differential effect of the salt reduction intervention across population groups with different levels of education either in terms of salt intake, knowledge or behaviors related to salt. While people with different educational levels might reasonably have been expected to gain different degrees of understanding about the intervention this was not apparent, and our findings suggest that the CRHI-SRS salt reduction program could be applied to good effect across rural Chinese populations regardless of their educational status. The broad-based uptake of the program likely reflects the care with which the program was designed and an explicit intent to design simple messages suited to rural populations where illiteracy remains a significant issue. Additionally we used a range of different ways to deliver those messages such that it was not just written materials provided but also public meetings integrated with performance, community leadership, and imagery.

The study suggests that positive effects of the program on knowledge and attitudes towards salt contributed to the salt reduction achieved. While a study done in Australia identified no association between knowledge, attitudes and behaviors towards salt and actual levels of salt intake [17] our context is somewhat different. In Australia most salt is hidden in processed foods and it is almost impossible for even highly motivated individuals to change their salt intake through behaviour. By contrast, in rural China, salt is mostly added by consumers during cooking and there is therefore much greater capacity for individuals to influence their own intake. For basically the same reasons the rural Chinese setting is also highly amenable to a significant impact from the use of salt substitute. The absence of any differential effect of price subsidization for the salt substitute across educational levels is another important observation for public health policy. With salt substitute a low cost commodity, there is a clear opportunity for government to introduce salt substitute nationwide with a high likelihood that the health benefits achieved would be distributed evenly across all sectors of the population.

Our study is one of the few that has explicitly addressed the impact of a salt reduction program across educational levels using a randomised and controlled design. A UK report evaluating a national salt reduction program involving the food industry, customer education and food labeling identified no inequity in the degree of salt reduction achieved across different socioeconomic groupings [18] although explicit reporting by educational level was not done.

The China non-communicable disease surveillance report in 2010 identified awareness of salt as a health issue amongst 56 % of rural Chinese and efforts to change behaviors related to salt amongst about one third [19]. Similarly low rates are likely in other resource poor settings around the world with a high burden of disease attributable to excess salt intake. Our program produced benefit amongst a poorly educated population with only limited application of resources and it is likely that tailoring the principles we employed to other developing country settings could produce important benefits elsewhere.

The present study is based on a large scale, well-designed, randomized controlled trial and provides a strong basis for the evaluations made here. We used 24-h urine collections which are the gold standard for estimating salt intake [20] and we appropriately focused on tests of heterogeneity across subgroups defined by educational level rather than the effects within each subgroup [21]. However, the trial was not designed *a priori* for the purpose of the present analyses and the power to detect the effects under investigation was limited. As with all post-hoc subgroup analyses there is also a risk of false positive findings consequent upon multiple testing although that did not arise as an issue here. Regardless, the conclusions from these analyses should be considered exploratory rather than confirmatory. About 90 % of our study population had less than 9 years of education which means that the results provided here provide rather limited insight into possible interactions at higher levels of education which may therefore have been missed.

## Conclusions

In conclusion, our study indicated equal effectiveness across educational groups of the community-based salt reduction program implemented with the CRHI-SRS. These data are encourageing because they suggest that an appropriately designed salt reduction program can importantly change salt intake regardless of people’s educational status.

## Abbreviations

CVD, cardiovascular disease; GEE, generalized estimating equations
